# Degradation of the Surface of Synthetic Layered Composites Due to Accelerated Ageing

**DOI:** 10.3390/ma18143342

**Published:** 2025-07-16

**Authors:** Cezary Strąk, Ewelina Kozikowska, Marcin Małek, Marcin Wachowski

**Affiliations:** 1 Building Research Institute, 1 Filtrowa St., 00-611 Warsaw, Poland; c.strak@itb.pl (C.S.); e.kozikowska@itb.pl (E.K.); 2 Institute of Civil Engineering, Faculty of Civil Engineering and Geodesy, Military University of Technology in Warsaw, 2. Gen. S. Kaliskiego, 00-908 Warsaw, Poland; 3 Faculty of Mechanical Engineering, Military University of Technology, 2 Gen. S. Kaliskiego 2, 00-908 Warsaw, Poland; marcin.wachowski@wat.edu.pl

**Keywords:** layered composites, aging, polyurethane, sport surfaces, UV radiation

## Abstract

This study investigates the effect of accelerated aging on the microstructure and surface properties of synthetic sports surfaces, with the goal of developing a more representative laboratory simulation method. Three common types of polyurethane-based sports surfaces were examined: (1) a dual-layer SBR base with a thin EPDM spray topcoat; (2) a single-layer EPDM surface with a smooth finish; and (3) a dual-layer “sandwich” structure with a rough EPDM upper layer. Samples were tested for slip resistance (PTV), abrasion resistance, and surface morphology using SEM, as well as surface roughness and tensile properties before and after aging. Method combining UV radiation and water spray was introduced and evaluated. Microstructural analysis with roughness measurements revealed surface degradation in all materials, with more extensive damage observed in the UV + spray cycle. Slip resistance results showed reduced performance in dry conditions and improved values in wet conditions post-aging. The single-layer EPDM surface demonstrated the highest initial dry PTV, while the dual-layer with spray had the lowest. After aging, all surfaces exhibited smaller differences between dry and wet performance but no longer met dry condition standards. These results may guide future revisions of performance testing standards and contribute to the development of safer, longer-lasting synthetic sports surfaces.

## 1. Introduction

Synthetic surface coatings for outdoor sports applications have become essential components of modern athletic infrastructure. These systems are engineered to combine safety, durability, and performance under variable environmental conditions. Typically constructed from ethylene propylene diene monomer (EPDM) or styrene–butadiene rubber (SBR) bound with polyurethane (PU) resins, synthetic sports surfaces offer a combination of elasticity, traction, energy return, and weather resistance critical for recreational and professional sports [1,2,3].

EPDM, a sulfur- or peroxide-vulcanized elastomer, is prized for its UV stability, long-term elasticity, and color retention, making it suitable for exposed top layers [1]. SBR, often sourced from recycled tires, contributes to the circular economy but is more prone to oxidative degradation and variability in composition [2]. Polyurethane binders act as the structural matrix, determining energy absorption, surface flexibility, and adhesion between filler particles [3].

The construction of sports coatings can follow several configurations, namely, single-layer, dual-layer, or sandwich systems, each tuned to achieve a balance between surface grip and shock attenuation. Smooth, trowel-finished EPDM surfaces often provide higher dry traction, whereas textured, sprayed coatings maintain performance under wet conditions [4,5]. Regardless of design, the surface layer remains the most critical for user interaction and is therefore the most susceptible to degradation.

Exposure to environmental stressors such as UV radiation, water (rain or condensation), temperature fluctuations, and mechanical wear can result in physical and chemical deterioration of the surface layer. Degradation typically manifests as surface chalking, color fading, embrittlement, cracking, and decreased elasticity, all of which compromise functional performance and safety [6,7]. UV radiation in particular initiates photochemical reactions that disrupt polymer chains, leading to oxidation and surface hardening [6]. Moisture, especially in porous or unsealed systems, exacerbates these effects by promoting hydrolysis and leaching of additives [5,8].

Artificial weathering methods are widely used to simulate years of natural exposure in a shortened timeframe. These tests often involve UV exposure in combination with moisture via condensation or water spray. While standardized methods such as EN 14836 and ISO 4892-3 guide these procedures, studies have shown significant discrepancies between accelerated and natural aging effects, particularly in polymer-based systems like EPDM and SBR [6,7]. Discrepancies in UV spectral output, humidity levels, and thermal cycles limit the reliability of artificial aging as a predictive tool. As a result, improved protocols tailored to the material-specific degradation pathways are needed for more accurate durability assessments.

One of the most pressing limitations of synthetic coatings is the loss of slip resistance over time, which poses direct safety risks to athletes. Surface hardening and smoothening reduce friction in dry conditions, while excessive textural degradation may lead to unpredictable performance under wet conditions [6]. Simultaneously, surface roughness plays a dual role—it enhances grip but can also trap water and dirt, negatively affecting drainage and hydrophobicity [4,9]. Abrasion resistance is another vital parameter, as coatings must withstand repeated foot traffic, ball impacts, and maintenance without substantial material loss. While harder aged surfaces may resist abrasion, this often comes at the cost of elasticity and comfort [3,9,10,11].

To mitigate these issues, research in materials engineering has focused on several directions. Incorporating stabilizers (e.g., UV absorbers, antioxidants) and nanofillers (e.g., graphene, titanium dioxide) has shown promise in improving photochemical stability and mechanical performance [1,3]. Adjusting binder formulations, particularly in PU systems, can enhance crosslinking efficiency and resistance to thermal degradation [8,10,12]. Additionally, surface coatings such as transparent sealants or pigmented overlays can function as sacrificial layers, shielding the structural base from direct exposure [4].

From a sustainability perspective, the use of recycled rubber (especially SBR) in surface systems supports waste reduction and cost savings. However, ensuring long-term durability of recycled-based materials remains a challenge due to their inconsistent quality and higher degradation rate [2]. Future material development must emphasize both performance and recyclability, ensuring that components can be recovered or safely disposed of at the end of their life cycle [5,13,14,15].

In the broader context of public infrastructure and health, surface coatings must meet evolving regulatory demands while providing a safe and high-performance environment for users. Performance indicators such as slip resistance, energy restitution, abrasion resistance, and color stability must be maintained throughout the product’s lifecycle. This is particularly relevant for facilities used by children, seniors, and athletes with high biomechanical demands [16,17].

Future research directions should focus on multi-scale degradation modeling, integrating physical aging data with chemical transformation kinetics to better predict material lifespan. Hybrid aging protocols combining natural and artificial exposure could offer more realistic evaluations. At the material level, novel elastomeric composites with tailored crosslink densities, hierarchical surface textures, and enhanced filler dispersion are promising avenues for improving long-term performance. Furthermore, advances in additive technologies and responsive coatings that adapt to environmental stimuli (e.g., temperature, humidity) may open new paths in smart surface design for sports applications.

## 2. Materials and Methods

Three types of synthetic sports surface systems were selected for the experimental program, each representing a commonly used configuration in outdoor sports infrastructure. All samples were manufactured in laboratory conditions using industry-standard materials supplied by commercial producers and prepared in accordance with the specifications defined in EN 14877:2014 for synthetic surfacing systems based on polyurethane and rubber granulates.

The systems differed in terms of structural configuration, surface finish, and material composition. To facilitate comparative analysis and maintain clarity throughout the study, the following sample designations were adopted:

- *Type A—“Spray-coated dual-layer system” (Sample A):*

**Figure 1:**
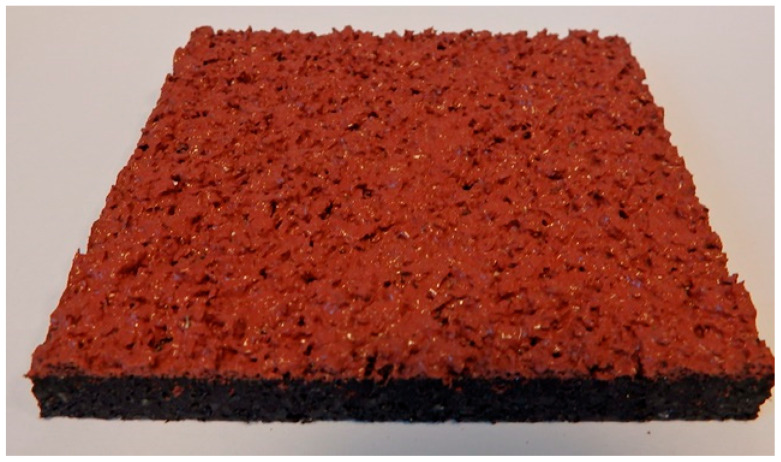
Two-layer (Sample A) surface made of SBR covered with a thin, rough spray-applied EPDM layer—a single-layer surface entirely made of EPDM, troweled smooth, and hereinafter referred to as the single-layer EPDM surface.

A two-layer structure (Figure 1) consisting of a base layer made of SBR (styrene–butadiene rubber) granules bound with polyurethane resin, topped with a fine-textured EPDM (ethylene propylene diene monomer) layer applied using a spray method. The top layer was formulated with colored EPDM granules (0.5–1.5 mm fraction) and aliphatic PU binder in a mass ratio of 1:0.2.

- *Type B—“Monolithic EPDM system” (Sample B):*

**Figure 2:**
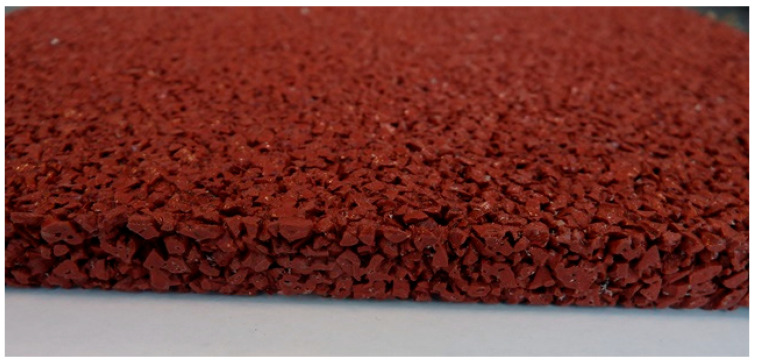
Single-layer (Sample B) surface entirely made of EPDM, troweled smooth.

A single-layer (Figure 2), homogeneous surface composed entirely of colored EPDM granules (1.0–3.5 mm fraction) uniformly mixed with polyurethane binder in a mass ratio of 1:0.25. The top layer was smoothed by troweling to create a compact, closed surface structure, often used in multipurpose sports courts.

- *Type C—“Sandwich system” (Sample C):*

**Figure 3:**
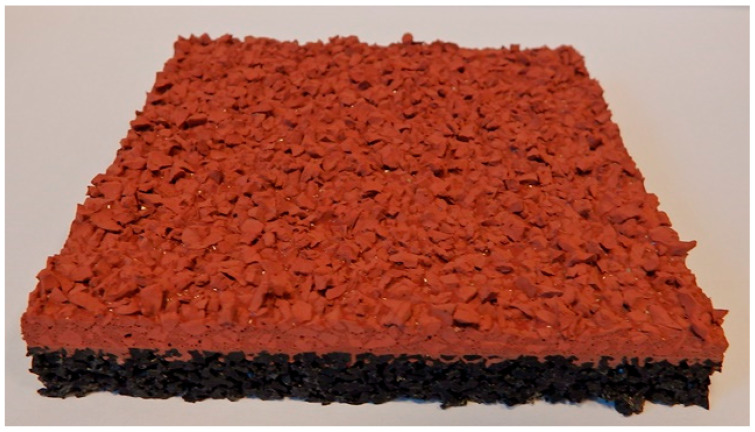
Two-layer surface (Sample C) consisting of a bottom SBR layer and a top EPDM layer, with a rough surface texture.

A dual-layer configuration (Figure 3) with an SBR base layer and a coarse-grained EPDM top layer (1.0–3.5 mm), both bound with polyurethane resin. The surface was finished using a troweling technique to achieve an open, textured finish optimized for spike resistance and traction under variable conditions. All polyurethane resins used in the surface systems were moisture-curing, solvent-free, one-component binders formulated for outdoor application. Both SBR and EPDM rubber fractions were characterized by stable granulometry and compliant with the chemical purity requirements of EN 14877:2014.

### Sample Dimensions and Conditioning

Rectangular slabs of each system were fabricated in metal molds to a thickness range 10 ÷ 15 mm, consistent with standard practice for performance testing of sports surfaces. After mixing and pouring, samples were cured under ambient laboratory conditions (23 ± 2 °C, 50 ± 5% RH) for a minimum of 7 days prior to testing or exposure. All samples were visually inspected to confirm the absence of voids, delaminations, or surface irregularities. To ensure consistency across test procedures, each surface type was replicated in a batch of at least six panels, allowing for statistical evaluation of mechanical and physical parameters both before and after artificial aging. Samples were labeled and stored under controlled conditions until testing.

## 3. Testing Methods

The microstructure of synthetic sports surfaces was investigated using a scanning electron microscope model Sigma 500 VP (Carl Zeiss Microscopy GmbH, Köln, Germany) with cold field emission enabling high resolution at low accelerating voltage. Investigations were carried out at an excitation electron beam accelerating voltage of 5 KeV, using an SE detector on samples sputtered with a gold layer. All observations were carried out at magnifications of 100, 200×, and 500×. The microstructure of the synthetic sports surfaces was observed in its initial state and after two different use cycles.

To comprehensively evaluate the performance characteristics of synthetic sports surface systems, a series of standardized laboratory tests were carried out on both aged and unaged specimens. The investigations included tensile testing, surface roughness measurements, slip resistance evaluation, and abrasion resistance analysis. All procedures were performed under controlled laboratory conditions and followed relevant European and international standards.

Tensile properties, including tensile strength and elongation at break, were determined in accordance with the PN-EN 12230:2005 standard, which applies to flexible polymeric materials. Tests were conducted using a ZWICK universal testing machine (Ulm, Germany) equipped with a 10 kN load cell, pneumatic grips, and an optical extensometer. Dumbbell-shaped specimens were prepared from each type of surface, with a gauge length of 150 mm, a width of 40 mm, and a thickness ranging from 10 to 15 mm, depending on the sample structure. Each specimen was conditioned for 24 h at 23 ± 2 °C and 50 ± 5% relative humidity prior to testing. The specimens were stretched at a crosshead speed of 50 mm/min until rupture, and both tensile strength and elongation at break were recorded. Five replicates were tested for each surface type and aging condition.

Surface roughness was analyzed using a Keyence VHX-7100 digital 3D microscope (Osaka, Japan), which enables non-contact profilometric measurement of microtopography. Square samples with dimensions of 50 mm × 50 mm were prepared from the top layer of each surface type, both before and after artificial aging. Each specimen was scanned at three distinct locations using a VH-Z100UR objective (Keyence Corporation, Osaka, Japan) (magnification range: 100–1000×). The following roughness parameters were evaluated according to ISO 4287:1997: arithmetic average roughness (Ra), maximum height of profile (Rz), and root mean square roughness (Rq). Measurements were taken under ambient lighting with integrated auto-focus and depth-composition mode, ensuring accurate imaging of surface features.

Slip resistance was assessed using the pendulum test method, as described in PN-EN 13036-4:2011. A Stanley Portable Skid Resistance Tester fitted with a rubber slider (IRHD 55) was used to simulate foot strike dynamics on the synthetic surface. The measurements were conducted in both dry and wet conditions, with water evenly applied to the test area during wet trials. For each test surface, square slabs with dimensions of 500 mm × 500 mm were used to allow proper pendulum swing. Five valid readings were obtained from each sample, and the mean Pendulum Test Value (PTV) was calculated.

Abrasion resistance was evaluated using the Taber rotary abrasion test in accordance with PN-EN ISO 5470-1:2017-02. Specimens with a diameter of 108 mm were punched from the surface material using a circular die. The tests were conducted on a Taber 5135 rotary abrader equipped with H18 abrasive wheels and a 1000 g load applied to each wheel. Each sample underwent 1000 revolutions, and mass loss was measured to the nearest 0.0001 g using a calibrated analytical balance. The results were compared with the maximum permissible value of 4.0 g, as stipulated in EN 14877:2014 for synthetic sports surfaces.

All tests described above were performed on both non-aged and artificially aged specimens. Accelerated aging was carried out using two distinct methods: the standard UV + condensation cycle specified in EN 14836, and an alternative UV + water spray protocol. The parameters of the aging process are describing below:

- Cycle 1 (EN 14836):
  - 4 h of UV exposure at an intensity of 0.80 W/(m^2^/nm), with a black panel temperature of 55 °C.
  - 2 h of condensation at a black panel temperature of 45 °C.

Total exposure time: 3000 h (2000 h of UV exposure, 1000 h of condensation)

- Cycle 2 (ISO 4892-3):
  - 5 h of UV exposure at an intensity of 0.83 W/(m^2^/nm), with a black panel temperature of 50 °C.
  - 1 h of water spray without temperature control.

Total exposure time: 3000 h (2500 h of UV exposure, 500 h of water spray)

Although the results of aging are not presented here, all post-aging specimens were subjected to the same mechanical and surface property tests, allowing for a reliable comparison of pre- and post-exposure performance. Each measurement was conducted on a minimum of five specimens per condition to ensure repeatability and statistical reliability. All instruments were maintained and calibrated according to manufacturer specifications prior to testing. The combination of these methods provided an integrated assessment of the durability and functional behavior of synthetic sports surfaces under conditions simulating long-term environmental exposure.

## 4. Research Results

### 4.1. Microstructure Analysis

#### 4.1.1. Analysis of the Surface Microstructure of Synthetic Sports Surfaces in the Initial (As-Received) Condition

**Figure 4:**
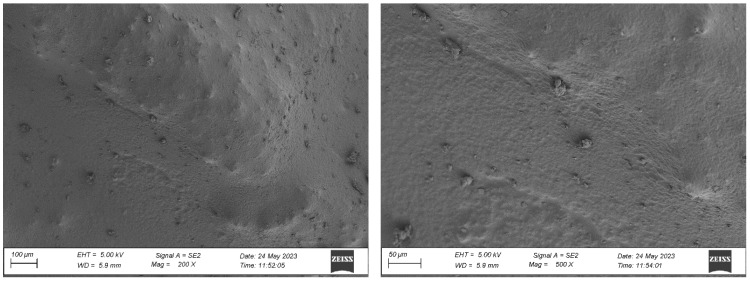
Surface microstructure of a two-layer polyurethane surface made of SBR covered with a thin, rough spray-applied EPDM layer at magnifications of 200× and 500× (Sample A).

**Figure 5:**
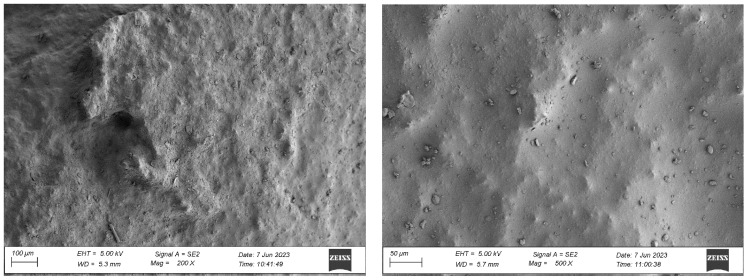
Surface microstructure of a single-layer polyurethane surface made of EPDM—troweled smooth at magnifications of 200× and 500× (Sample B).

**Figure 6:**
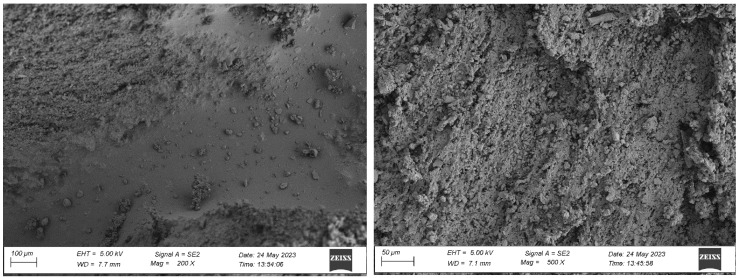
Surface microstructure of a two-layer polyurethane “sandwich” surface consisting of a bottom SBR layer and a top EPDM layer with a rough surface texture at magnifications of 200× and 500× (Sample C).

All three investigated surface (Figure 4, Figure 5 and Figure 6) types exhibited a uniform and continuous surface morphology with no visible structural defects, delaminations, or cracks. The specimens were visually homogeneous and presented no evidence of curing-related anomalies such as blistering, void formation, or edge detachment. The surface quality was consistent across each panel, which confirms the reproducibility of the preparation process and the compatibility of the polyurethane binder with the rubber granulate systems used. Minor surface irregularities in the form of small, localized inclusions or micro-residue traces were observed in some samples, likely originating from the manual processing phase, including molding, demolding, or surface finishing. These impurities, however, were superficial and did not affect the integrity or continuity of the coating.

For the spray-coated dual-layer surface (Sample A—Figure 4), a fine-textured and slightly granular finish was observed. The EPDM topcoat, applied via spray, resulted in a controlled micro-roughness characterized by the presence of exposed fine granules embedded in a thin film of binder. This configuration is commonly used to improve water drainage and maintain wet grip performance. The visual examination revealed no sign of granule loss, binder separation, or discoloration. The surface was cohesive, and the granulate was uniformly distributed, providing a consistent texture over the entire area.

In the case of the monolithic EPDM surface (Sample B) shown in Figure 5, the surface was smooth and compact, with a trowel-finished texture typical of high-friction court coatings. The visual inspection confirmed a tight, closed surface matrix, indicative of good compaction and even granule distribution. The absence of open pores or granule pull-out zones further validated the uniformity of the resin-to-rubber ratio and sufficient curing of the polyurethane matrix.

In contrast, the sandwich-type dual-layer surface (Sample C—Figure 6), composed of a lower structural layer made from recycled SBR and a top layer of coarse-grained EPDM, exhibited a clearly heterogeneous microstructure with a pronounced grainy surface profile. The surface texture was intentionally rough and open-pored, with exposed EPDM granules protruding from the binder matrix. This construction technique is characteristic of surfaces designed for spiked athletic shoes, where increased macro-roughness enhances traction and mechanical interlock. The granulate size distribution (1.0–3.5 mm) contributed to the irregular surface geometry, leading to microcavities between particles and visible texture transitions. Despite the intended roughness, no defects such as granule detachment, binder shrinkage, or void formation were detected, confirming proper material compatibility and adequate curing conditions.

Overall, the surfaces exhibited material-specific textural characteristics that were consistent with their design intent. The compactness of the monolithic EPDM, the fine-grained nature of the spray-coated system, and the coarse-grained, open structure of the sandwich-type surface together provided a diverse range of surface finishes, which could be expected to respond differently under environmental exposure and mechanical testing. These distinctions in initial surface morphology formed the basis for subsequent evaluations of microstructural changes and performance degradation due to artificial aging.

#### 4.1.2. Analysis of the Surface Microstructure of Synthetic Sports Surfaces After Aging Involving UV Exposure and Condensation (Cycle 1)

Exposure of synthetic sports surfaces to accelerated aging conditions results in pronounced alterations in the morphology of the functional (user-exposed) surface. The degradation mechanisms induced by a combined action of ultraviolet (UV) radiation and moisture condensation lead to distinct physical and microstructural transformations that are observable across all three tested surface types. These changes significantly affect the visual appearance, continuity, and integrity of the upper layer, indicating the progressive deterioration of the polymer matrix under environmental stressors.

**Figure 7:**
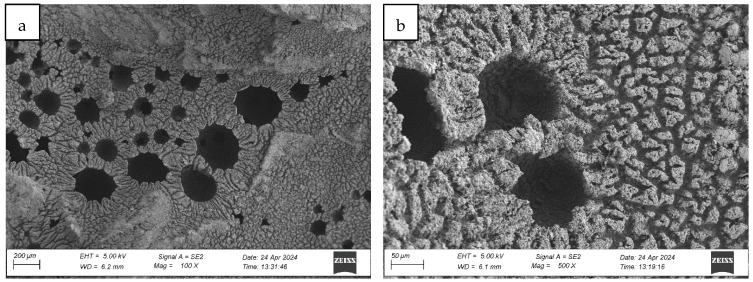
Surface microstructure of a two-layer polyurethane surface made of SBR covered with a thin, rough spray-applied EPDM layer after aging cycle 1 at magnifications of (**a**) 100× and (**b**) 500× (Sample A).

Post-aging inspection using high-resolution microscopy revealed the presence of surface melting phenomena, evident in the formation of regularly distributed pits, craters, and depressions within the polymer-rich top layer (cf. Figure 7, Figure 9 and Figure 12). These features are characteristic of thermal softening and localized photothermal degradation, where prolonged UV exposure compromises the molecular structure of the elastomer, particularly in binder-rich regions, leading to thermoplastic-like flow and surface collapse.

**Figure 8:**
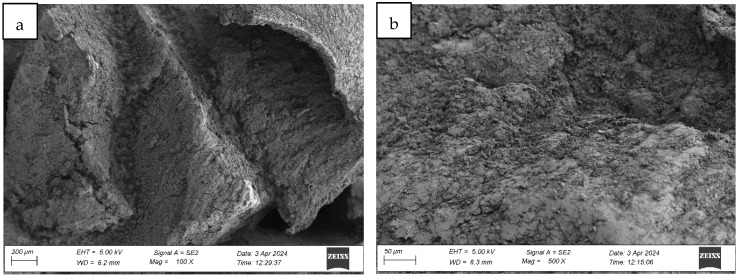
Surface microstructure of a single-layer surface made of EPDM—troweled smooth after aging cycle 1 at magnifications of (**a**) 100× and (**b**) 500× (Sample B).

**Figure 9:**
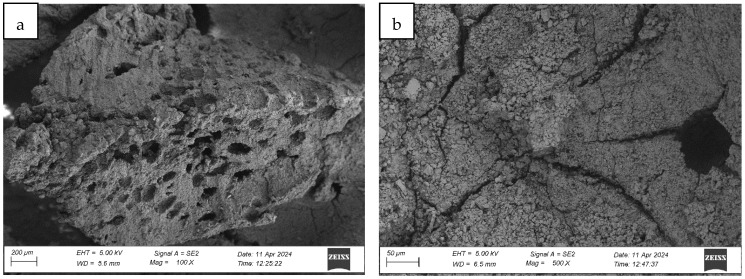
Surface microstructure of a two-layer polyurethane “sandwich” surface consisting of a bottom SBR layer and a top EPDM layer with a rough surface texture after aging cycle 1 at magnifications of (**a**) 100× and (**b**) 500× (Sample C).

In addition to localized melting, surface cracking was observed in the form of longitudinal fissures, microcracks, and interconnected crack networks (cf. Figure 7b, Figure 8b and Figure 9a). These discontinuities often originated in weakened zones between granules or at binder-matrix interfaces and extended progressively under cyclic thermal and hygroscopic stress. In some areas, cracks exhibited widening and deepening, evolving into branched cracking patterns that suggest ongoing embrittlement and loss of cohesion within the surface layer. The crack propagation pathways indicate that the material experienced not only photochemical damage but also mechanical fatigue due to differential expansion and contraction during the aging cycle.

The once uniform and compact microstructure observed in non-aged materials was visibly disrupted. The outermost surface lost its original smoothness and homogeneity, becoming visibly rough, porous, and fragmented. This is consistent with polymer oxidation and microstructural collapse, where UV-induced chain scission and oxidative crosslinking reduce flexibility and render the surface brittle. The degradation process further facilitates the detachment of fine particles or binder flakes, contributing to a dusty or eroded appearance.

The severity and morphology of the damage varied slightly depending on the surface system. While monolithic EPDM surfaces retained a relatively cohesive matrix with shallow surface melting, spray-coated and sandwich-type systems exhibited more irregular and deeper degradation features, particularly in areas with exposed granules and lower binder content. This suggests that the microtexture and granulate arrangement influence the thermal and photochemical stability of the surface, possibly by modifying local reflectance, moisture retention, and thermal conduction properties.

Collectively, the observations confirm that synthetic sports surfaces undergo marked degradation under the influence of UV radiation and condensation cycles, even under controlled laboratory conditions. The loss of structural integrity and functional microtexture in the top layer may have critical implications for long-term surface performance, particularly in regard to slip resistance, abrasion behavior, and mechanical flexibility. These findings reinforce the necessity of evaluating aging resistance not only through bulk mechanical properties but also via detailed morphological and microstructural analysis, which can reveal early signs of material fatigue and impending failure.

#### 4.1.3. Analysis of the Surface Microstructure of Synthetic Sports Surfaces After Aging Involving UV Exposure and Water Spraying (Cycle 2)

Exposure of synthetic sports surfaces to accelerated aging involving ultraviolet (UV) radiation in combination with water spray (“rain simulation”) induces substantial and progressive degradation of the outer polymer layer. Post-aging surface analysis across all three tested systems revealed extensive morphological transformations, consistent with advanced photothermal and hydrolytic degradation mechanisms.

**Figure 10:**
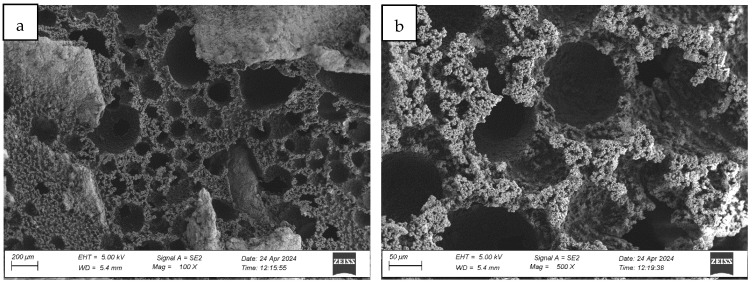
Surface microstructure of a two-layer polyurethane surface made of SBR covered with a thin, rough spray-applied EPDM layer after aging cycle 2 at magnifications of (**a**) 100× and (**b**) 500×.

**Figure 11:**
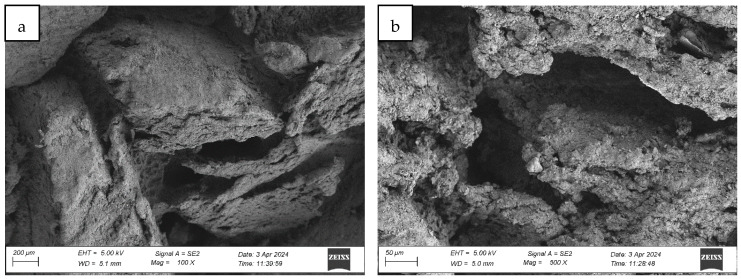
Surface microstructure of a single-layer polyurethane surface made of EPDM—troweled smooth after aging cycle 2 at magnifications of (**a**) 100× and (**b**) 500×.

**Figure 12:**
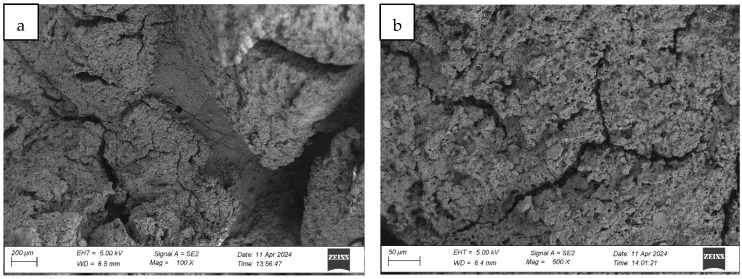
Surface microstructure of a two-layer polyurethane “sandwich” surface consisting of a bottom SBR layer and a top EPDM layer with a rough surface texture after aging cycle 2 at magnifications of (**a**) 100× and (**b**) 500×.

Macroscopic and microscopic evaluations confirmed the presence of surface melting phenomena, which manifested as regularly distributed pits and depressions, accompanied by numerous fine surface scratches and linear marks (cf. Figure 10, Figure 11 and Figure 12). These alterations are indicative of localized polymer flow and matrix disintegration, resulting from prolonged exposure to UV radiation and simultaneous mechanical and thermal stress generated by water impingement. The sprayed water, applied cyclically during the aging protocol, appears to intensify thermal gradients and surface fatigue, promoting superficial breakdown and binder erosion.

In addition to melting and superficial etching, deep surface cracking was observed in all systems. The cracks developed as longitudinal fissures and transverse fractures, many of which intersected and expanded into branched crack networks that compromise the mechanical cohesion of the surface layer. The propagation and densification of these cracks across larger areas mirror the degradation patterns observed in UV + condensation aging, yet the extent and intensity of damage were significantly more severe in the UV + spray condition. The recurring cycles of surface wetting and drying, combined with thermal loading, likely accelerated the loss of flexibility and enhanced embrittlement within the elastomer matrix. Upon UV exposure, polyurethane binders—particularly those containing aromatic moieties—undergo photochemical chain scission and radical formation. These photo-generated free radicals can react with atmospheric oxygen, initiating a cascade of oxidation reactions. This includes the formation of hydroperoxides, alcohols, ketones, and carbonyl groups, which compromise the polymer’s molecular integrity. The resultant oxidative crosslinking and chain cleavage lead to embrittlement, surface cracking, discoloration, and loss of flexibility. These transformations manifest as the fine scratches, micro-pits, and surface discontinuities observed in Figure 10, Figure 11 and Figure 12, particularly under the more aggressive UV + water spray aging conditions. This mechanism is consistent with established degradation pathways in polyurethanes, as detailed in [6,8], where photolytic breakdown is coupled with hydrolytic effects in the presence of moisture to accelerate surface oxidation and binder erosion.

The originally compact and homogeneous surface texture was entirely disrupted, with the polymer matrix exhibiting loss of cohesion, increased porosity, and a roughened, granular appearance. The fine scratches and erosion patterns suggest enhanced mechanical abrasion due to droplet impact, compounded by photochemical oxidation of exposed binder surfaces. Surface integrity was further compromised by the selective detachment of binder-rich regions, creating microscale cavities and textural inhomogeneities.

Comparative microstructural analysis confirmed that UV + spray aging induces a higher degree of surface degradation than the conventional UV + condensation method. This was particularly evident in the greater depth and density of cracks, more extensive melting zones, and the higher frequency of microstructural discontinuities observed under high-magnification imaging. These findings point to a more aggressive aging mechanism, likely due to the dynamic interaction between UV radiation, thermal stress, and the mechanical action of water droplets.

In summary, aging with UV and water spray leads to intensified morphological damage, resulting in the breakdown of surface smoothness, loss of protective top-layer continuity, and the development of widespread cracking and deformation. The pronounced deterioration observed under these conditions suggests that UV + spray protocols may more closely simulate real-life exposure scenarios, particularly in environments with alternating sun and rainfall cycles, and may serve as a more stringent benchmark for evaluating the long-term durability of synthetic sports surface systems.

### 4.2. Slip Resistance

#### 4.2.1. Slip Resistance Results in the Initial (Unaged) State

Table 1 and Table 2 present the slip resistance test results under dry and wet conditions for surfaces not subjected to aging.

Summarizing the slip resistance results obtained under dry conditions, the monolithic EPDM surface demonstrated the highest resistance to slipping, achieving a peak value of 83 PTV units. This superior performance is likely attributed to the maximized contact area between the test slider and the smooth, compact surface finish of the EPDM layer. The high degree of surface compaction, combined with the absence of macropores or granule protrusions, promotes uniform frictional interaction during pendulum testing.

In contrast, the lowest slip resistance values were recorded for the spray-coated dual-layer surface, which reached 75 PTV units under dry conditions and only 51 units under wet conditions. These results suggest that the fine-textured, open-structured surface created by spray application provides a reduced contact area for the slider, diminishing frictional resistance—particularly when surface moisture is introduced. The microtexture created by discrete EPDM particles suspended in a thin binder film may trap water and create a lubricating effect, further reducing grip.

Among the three tested surface types, the greatest drop in slip resistance between dry and wet conditions was observed for the monolithic EPDM surface, which exhibited a decrease of 27 PTV units. This significant reduction may be attributed to changes in surface hydrophobicity, combined with the potential formation of a thin water film on the smooth surface that temporarily interferes with contact friction. Despite this decline, the surface still maintained acceptable slip resistance within the normative wet condition range.

Overall, the monolithic EPDM surface provided the most favorable anti-slip performance, especially under dry conditions. These findings underscore the importance of surface finishing methods—specifically, whether the surface is trowel-smoothed or spray-applied—in determining functional slip behavior. The texture, granule distribution, and binder exposure all contribute critically to frictional interactions between users and the surface.

When the results were evaluated in light of the requirements defined in PN-EN 14877:2014, which specifies acceptable ranges of 55–110 PTV under wet conditions and 80–110 PTV under dry conditions, compliance varied across the materials. Under dry conditions, only the monolithic EPDM surface met the minimum threshold of 80 PTV. The sandwich-type and spray-coated systems fell below this requirement, indicating a potential safety concern in dry environments, particularly for high-performance athletic use.

Under wet conditions, two of the three surfaces—monolithic EPDM and sandwich-type—remained within the acceptable range. However, the spray-coated surface failed to meet the normative minimum of 55 PTV, raising concerns about its use in outdoor facilities where surface wetness is expected due to rain or irrigation. This suggests that while spray coatings may offer certain economic or installation advantages, their functional limitations under wet conditions must be carefully considered, especially in contexts where user safety and regulatory compliance are critical.

#### 4.2.2. Slip Resistance Results After UV + Condensation Aging Cycle (Cycle 1)

Table 3 and Table 4 present the slip resistance test results under dry and wet conditions for surfaces subjected to aging (Cycle 1: UV + condensation).

#### 4.2.3. Slip Resistance Results After UV + Water Spray Aging Cycle (Cycle 2)

Table 5 and Table 6 present the slip resistance test results under dry and wet conditions for surfaces subjected to aging (Cycle 2: UV + water spray).

**Figure 13:**
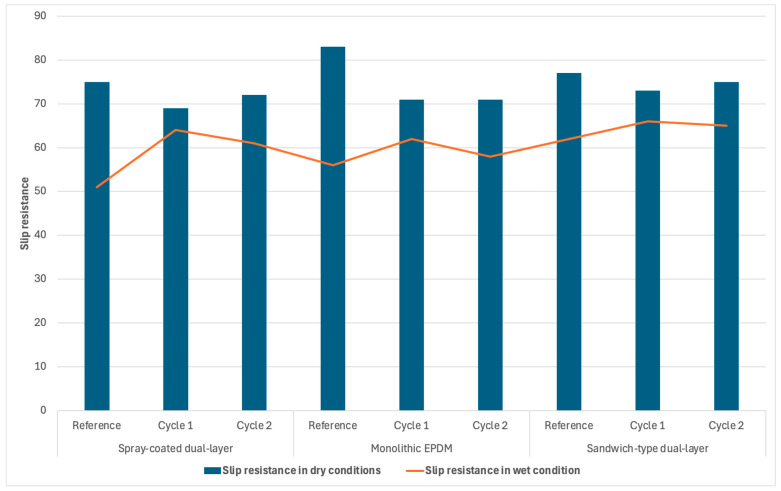
Graphical summary of slip resistance measurements before and after aging using both methods.

Table 3 and Table 4 present the slip resistance test results under dry and wet conditions for the three tested synthetic sports surfaces after aging using Cycle 1: UV + condensation; while Table 5 and Table 6 summarize the results for surfaces subjected to Cycle 2: UV + water spray. Table 7 and Figure 13 provides a comparative overview of slip resistance values before and after aging using both methods.

Following Cycle 1 (UV + condensation), a general decrease in slip resistance under dry conditions was observed across all surface types. The monolithic EPDM surface retained the highest dry slip resistance (Table 3), though its value dropped slightly compared to the unaged state. The spray-coated dual-layer surface exhibited the lowest values in both dry and wet conditions (Table 3 and Table 4), confirming its greater susceptibility to surface degradation and loss of functional microtexture. The sandwich-type surface demonstrated moderate performance, with slip resistance values decreasing in the dry state (Table 3) but remaining within acceptable limits in the wet state (Table 4).

Under wet conditions, all surfaces showed reduced variability between samples and generally retained compliance with PN-EN 14877:2014, which specifies a minimum of 55 PTV. The exception was the spray-coated surface (Table 4), which once again failed to meet the standard, suggesting insufficient performance in humid environments after aging via condensation.

The results obtained after Cycle 2 (UV + water spray) (Table 5 and Table 6) revealed more pronounced surface degradation effects. Under dry conditions, slip resistance decreased for all materials, with the spray-coated system again exhibiting the lowest values (Table 5). Notably, the monolithic EPDM (Table 5) and sandwich-type surface (Table 5) experienced only marginal declines, maintaining values near the normative threshold of 80 PTV.

In wet conditions, the monolithic EPDM (Table 6) and sandwich-type surface (Table 6) remained compliant with PN-EN 14877:2014. However, the spray-coated surface (Table 6) again fell below the minimum required PTV value, reinforcing the earlier observation of its limited durability under water-assisted photothermal aging.

Table 7 provides a direct comparison of all surface types before and after both aging cycles. The monolithic EPDM surface consistently exhibited the best anti-slip properties, particularly under dry conditions, while also maintaining acceptable performance when wet. In contrast, the spray-coated system consistently showed the poorest slip resistance, both in initial and post-aging states, particularly under wet conditions. The sandwich-type surface provided a balanced performance profile, with moderate degradation across both cycles and acceptable values under most test conditions.

### 4.3. Abrasion Resistance

#### Abrasion Resistance Results of the Surfaces in Their Initial (Unaged) State and After Aging

**Figure 14:**
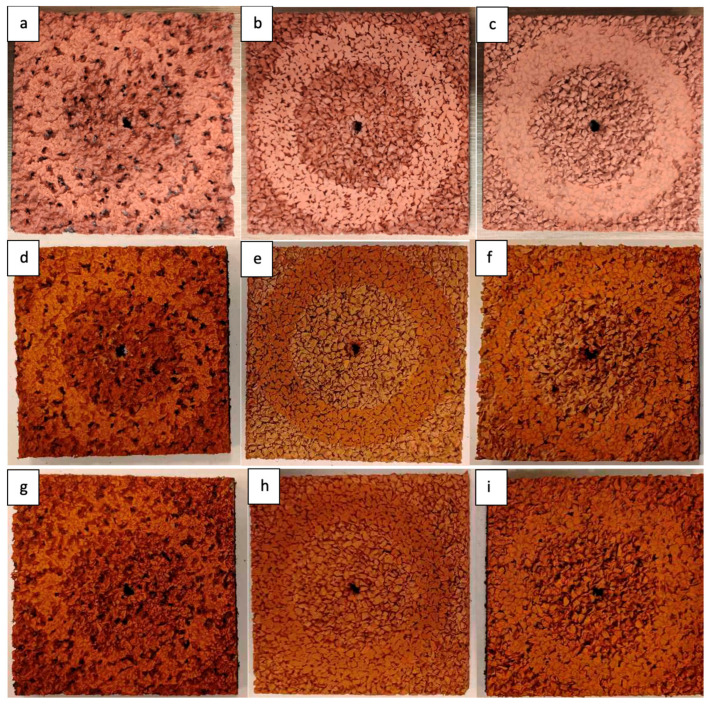
View of the unaged surface samples after abrasion resistance testing: (**a**–**c**); after being subjected to aging (UV + condensation) after abrasion resistance testing: (**d**–**f**); and after being subjected to aging (Cycle 2: UV + water spray) after abrasion resistance testing: (**g**–**i**). Dual-layer surface with SBR base and thin spray-coated EPDM top layer: (**a**,**d**,**g**); single-layer surface entirely made of EPDM: (**b**,**e**,**h**); dual-layer sandwich-type surface with SBR base and EPDM top layer: (**c**,**f**,**i**).

**Figure 15:**
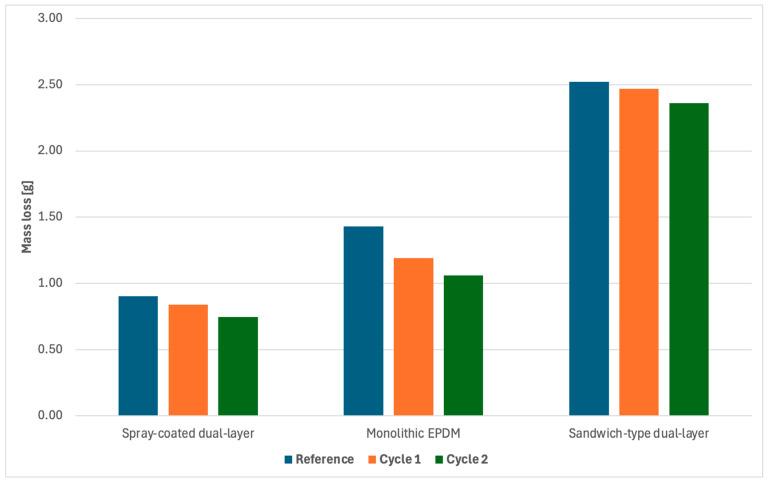
Graphical summary of abrasion resistance test results for synthetic sports surfaces.

The results are presented in Table 8 and Figure 14 and Figure 15, while the samples after abrasion are shown in the photograph below.

The abrasion resistance of the synthetic sports surfaces was evaluated using the Taber abrasion test in accordance with PN-EN ISO 5470-1:2017-02. The test results are summarized in Table 8 and Figure 15, providing a comprehensive overview of the material’s resistance to wear under controlled conditions.

In general, the monolithic EPDM surface demonstrated the highest abrasion resistance, with the lowest mass loss observed across all tested samples. This surface retained its structural integrity well, even after prolonged exposure to abrasion, which is likely attributed to the compactness and uniformity of the single-layer EPDM system. The smooth, troweled finish provided a dense matrix with minimal exposed voids, offering strong protection against mechanical wear.

In contrast, the spray-coated dual-layer surface exhibited the highest mass loss and the lowest abrasion resistance among all the samples. The thin spray layer of EPDM on the SBR base was more susceptible to wear, likely due to the looser texture and lower binder content. The surface exhibited more pronounced deterioration, with visible erosion and granule pull-out occurring during the abrasion process. This indicates that while the spray-coated system may offer certain performance benefits in terms of texture or water drainage, its ability to withstand mechanical stress is limited.

The sandwich-type dual-layer surface displayed moderate abrasion resistance, with mass loss values falling between those of the monolithic EPDM and the spray-coated dual-layer system. The coarse EPDM top layer combined with the SBR base provided a surface with sufficient resistance to wear, although localized wear patterns were more noticeable in areas where the granules were more exposed. The surface maintained its performance better than the spray-coated system but was not as durable as the monolithic EPDM.

After aging, under both Cycle 1 (UV + condensation) and Cycle 2 (UV + water spray), all surfaces experienced a slight increase in mass loss, indicating reduced abrasion resistance as a result of environmental degradation. This effect was most pronounced in the spray-coated dual-layer surface, which exhibited significant changes in texture and a higher rate of mass loss compared to unaged samples. The monolithic EPDM surface retained relatively good performance even after aging, demonstrating its durability and resistance to surface degradation under UV and moisture exposure.

### 4.4. Tensile Strength Test

**Figure 16:**
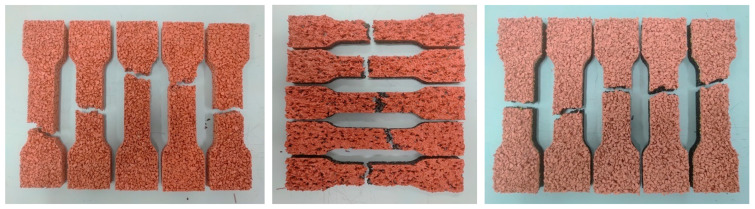
All tested samples after tensile strength measurement.

The tensile strength and elongation at break of the synthetic sports surface samples were evaluated to assess the impact of accelerated aging on their mechanical performance. The results, summarized in Table 9 and Figure 16, demonstrate material-specific responses to environmental degradation under two distinct artificial aging protocols—UV radiation combined with condensation (Cycle 1) and UV radiation combined with water spray (Cycle 2).

In the unaged state, the monolithic EPDM surface exhibited the highest tensile strength of 0.93 MPa and elongation at break of 52.4%, confirming its superior mechanical integrity. These values reflect the homogenous, compact structure of the EPDM material, which provides a well-distributed stress response under tensile loading. After aging, tensile strength slightly increased under Cycle 1 (0.98 MPa), possibly due to post-curing effects or UV-induced surface hardening, while elongation remained relatively stable (53.3%). However, exposure to the more aggressive Cycle 2 resulted in a drop in tensile strength to 0.89 MPa and a decrease in elongation to 46.2%, suggesting progressive embrittlement and reduced ductility caused by combined UV and water spray degradation.

The spray-coated dual-layer system, in contrast, showed the lowest elongation across all conditions, beginning with 50.9% in the reference state and dropping to 38.7% after Cycle 1. Although the tensile strength remained relatively unchanged (approx. 0.83–0.84 MPa), the significant loss in flexibility indicates substantial deterioration of the binder–granulate matrix. After Cycle 2, a partial recovery in elongation (41.6%) was observed, but this may be attributed to surface-level softening rather than true structural resilience.

The sandwich-type dual-layer system recorded the lowest tensile strength in the reference state (0.57 MPa), a result consistent with its open-pored, coarse-grained surface design, which provides less structural continuity. However, after exposure to UV aging cycles, a notable increase in tensile strength was observed, rising to 0.82 MPa after Cycle 1 and slightly decreasing to 0.75 MPa after Cycle 2. This strengthening effect could stem from UV-induced crosslinking within the polyurethane binder, but it occurred at the expense of flexibility, as evidenced by elongation values fluctuating between 37.8% and 45.7%.

Overall, these findings confirm that artificial aging influences not only the surface condition of synthetic sports systems but also their internal mechanical behavior. While slight improvements in tensile strength may occur due to hardening effects, the simultaneous loss of elasticity, particularly in complex multi-layer systems, poses long-term concerns regarding brittleness and crack propagation under dynamic loading.

### 4.5. Surface Roughness Analysis

Complementing the tensile strength results, surface roughness analysis provided additional insight into microstructural transformations induced by aging. Surface roughness parameters were obtained using a 3D digital microscope, focusing primarily on the vertical surface deviation metric S_a_, which reflects the extent of topographical changes caused by degradation (Table 10). Surface roughness analysis was revealed also by 3D reconstruction maps, shown in Table S1.

Surface roughness measurements, expressed as the arithmetic mean height (Sa), provided detailed insights into the microtopographical degradation of synthetic sports surfaces under accelerated aging conditions. The initial roughness of the spray-coated dual-layer surface was measured at 431.08 µm, reflecting a moderately textured surface formed by fine EPDM granules spray-applied over an SBR base. After exposure to Cycle 1 (UV + condensation), the Sa value increased to 543.65 µm, indicating surface destabilization likely caused by binder shrinkage, microcracking, and localized granule exposure. This suggests that UV-induced photodegradation combined with thermal cycling weakens the polymer matrix, leading to surface roughening. In contrast, Cycle 2 (UV + water spray) resulted in a Sa reduction to 367.84 µm. This decrease may be attributed to the abrasive effect of water droplets eroding the most prominent surface features, potentially collapsing microcracks and washing away loosely adhered particles. While the surface appeared visually smoother, the reduction in Sa likely reflects a loss of functional microtexture, which may impair slip resistance and water drainage capabilities.

The monolithic EPDM surface, characterized by the highest initial roughness (Sa = 746.86 µm), exhibited an increase to 831.32 µm after Cycle 1. This change reflects the formation of microdefects and stiffening of the polymer network under UV exposure, which disrupted the original compact structure. The relatively high starting roughness, despite the trowel-smoothed finish, is attributable to the inherent variability of large EPDM granules distributed within the polyurethane binder. Following Cycle 2, however, the Sa value decreased substantially to 521.53 µm, suggesting significant erosion of surface features. The interaction of UV degradation with mechanical water spray likely contributed to the breakdown of binder-rich zones, removal of weakened material, and general flattening of the surface. Although this process reduced the surface roughness, it may have compromised tactile grip and accelerated functional wear, indicating a trade-off between aesthetic leveling and mechanical degradation.

The sandwich-type dual-layer surface initially the smoothest of the three in terms of Sa (287.90 µm), displayed a pronounced increase in roughness to 426.54 µm after Cycle 1. This indicates the onset of surface fragmentation, granule boundary separation, and microcavity formation within the coarse EPDM top layer. The open-pore configuration, while beneficial for traction under spiked footwear, is particularly susceptible to UV-induced binder decay and intergranular cracking. After Cycle 2, the Sa value declined to 329.45 µm, a reduction likely caused by the smoothing of rough edges and partial removal of exposed granules. This implies that the dynamic combination of UV radiation and cyclic water impingement not only accelerates material fatigue but also mechanically alters the surface profile by rounding protrusions and collapsing weak structural elements.

Taken together, these results demonstrate that accelerated aging processes affect surface roughness in different ways depending on the material composition, surface structure, and type of environmental stressor. UV radiation combined with condensation (Cycle 1) tends to increase surface roughness by promoting crack formation, binder contraction, and granule exposure. In contrast, UV radiation combined with water spray (Cycle 2) leads to partial smoothing of the surface through erosion and particle detachment, even as underlying damage continues to accumulate. Notably, the changes in Sa values correlate with previously observed degradation phenomena such as microcracking, surface melting, and granule loosening.

## 5. Discussion

The results obtained from the abrasion resistance tests, as presented in Table 8, demonstrate significant variations in the wear performance of the three types of synthetic sports surfaces under study. The surfaces were subjected to both unaged and aged conditions, including accelerated aging via two distinct protocols: UV + condensation (Cycle 1) and UV + water spray (Cycle 2). These results are crucial for understanding how environmental stressors such as UV radiation, moisture, and mechanical wear affect the long-term durability of synthetic sports surfaces.

In the initial unaged state, the monolithic EPDM surface exhibited the highest abrasion resistance, with the lowest mass loss among the three materials. This is consistent with its compact structure and smooth finish, which provide strong resistance to mechanical wear. The smooth, troweled surface offers minimal exposed voids, leading to uniform frictional interaction during abrasion testing, which contributes to its superior performance. These results align with findings from previous studies, such as those by Yuan et al. [1], who reported that monolithic elastomeric systems, particularly those based on EPDM, exhibit strong abrasion resistance due to their dense polymer matrix and minimal filler content. Conversely, the spray-coated dual-layer system demonstrated the highest mass loss, indicating lower abrasion resistance. The thin spray layer of EPDM on the SBR base likely contributes to this reduced durability. The looser texture and lower binder content in the spray-coated system make it more susceptible to wear. This finding supports research by Azizi et al. [3], who noted that spray-coated surfaces are more prone to deterioration under mechanical stress due to their reduced structural cohesion and increased susceptibility to surface erosion.

The sandwich-type dual-layer system, consisting of an SBR base and a coarse-grained EPDM top layer, showed moderate abrasion resistance. While it did not outperform the monolithic EPDM surface, it did maintain a better performance than the spray-coated system. The coarse-grained texture of the EPDM top layer, while offering good traction, also exposed more granular surface features to mechanical wear, leading to localized abrasion. This result is consistent with the study by Al Maamori and Kateeb [9], which highlighted that the open-pore structure of certain synthetic surfaces, while beneficial for traction, often compromises abrasion resistance.

After undergoing Cycle 1: UV + condensation, all surfaces showed an increase in mass loss, indicating a reduction in abrasion resistance due to environmental degradation. However, the monolithic EPDM surface still maintained relatively low mass loss compared to the other systems, confirming its durability under aging. The spray-coated dual-layer system exhibited the greatest increase in mass loss, which can be attributed to the softening and surface degradation caused by the UV radiation and condensation cycles. The higher rate of material breakdown in this system is consistent with findings from Hong et al. [8], who demonstrated that UV exposure accelerates the degradation of polyurethane-based systems, particularly those with thinner, less cohesive surface coatings. The sandwich-type dual-layer system showed moderate degradation after aging, with a slight increase in mass loss compared to its unaged state. While it exhibited better resistance than the spray-coated system, its coarse texture still made it susceptible to wear, albeit to a lesser extent than the spray-coated variant. This result highlights the importance of the texture-grain structure and material composition in determining the abrasion resistance of sports surfaces.

The Cycle 2: UV + water spray results revealed more severe degradation effects than those of Cycle 1, especially in the spray-coated dual-layer system. The repeated cycles of UV radiation and water impingement led to more pronounced surface softening, cracking, and erosion. This was particularly evident in the spray-coated system, which exhibited significant changes in surface morphology, including the formation of craters, microcracks, and deeper erosion zones. These changes suggest that water spray exacerbates the thermal degradation caused by UV exposure, accelerating polymer chain scission and binder erosion. This finding is consistent with the work of Wachtendorf et al. [5] and Małek et al. [13], who found that water spray combined with UV exposure significantly increases the wear rate of polymer-based materials.

The monolithic EPDM surface demonstrated the least degradation under Cycle 2, maintaining its structural integrity and showing only minor increases in mass loss. While abrasion resistance decreased somewhat, the surface still performed better than the spray-coated system. This resilience could be attributed to the higher density and greater chemical stability of the monolithic EPDM, which resists both UV-induced oxidation and mechanical wear. Similar findings were reported by Tagliabue et al. [6] and Szymański et al. [18], who noted that single-layer EPDM systems tend to retain better abrasion resistance compared to multi-layered systems when subjected to UV exposure and moisture cycles.

The sandwich-type system exhibited moderate degradation, but the increase in mass loss was less significant than that observed in the spray-coated system. The rough surface texture, designed for improved traction, contributed to higher wear rates under both aging conditions. However, the overall performance remained acceptable for most applications. This aligns with previous studies by Andena et al. [7], who reported that sandwich systems maintain relatively good performance in terms of both abrasion resistance and traction despite the challenges posed by environmental degradation.

The observed degradation trends in this study are consistent with several other investigations into the aging behavior of synthetic sports surfaces. For instance, Liu et al. [2] studied the chemical degradation of EPDM in acidic environments and found that crosslinking density and material formulation significantly impacted the abrasion resistance of the material. Similarly, Salimi et al. [4], Wojcik et al. [19] and Pawlak et al. [20] highlighted that recycled rubber in synthetic surfaces, like the SBR used in sandwich-type systems, exhibits a higher degradation rate than virgin EPDM, which could explain the observed differences in wear performance.

Furthermore, Kim et al. [10] and Kaczmarek et. al. [21] explored the mechanical behavior of rubber-based layers and found that surface roughness and texturing influence both traction and abrasion resistance in synthetic sports surfaces. Their findings align with our observations that rougher, textured surfaces like the sandwich-type system experience higher abrasion but maintain good traction, while smoother surfaces like monolithic EPDM provide superior abrasion resistance.

## 6. Conclusions

This study assessed the durability of three common synthetic sports surface systems: monolithic EPDM, spray-coated dual-layer, and sandwich-type dual-layer. Mechanical, morphological, and microstructural properties were analyzed before and after accelerated aging using two protocols—Cycle 1 (UV + condensation) and Cycle 2 (UV + water spray)—to simulate environmental degradation.

Cycle 2 proved more damaging, resulting in reduced tensile properties, decreased wet slip resistance, surface hardening, and smoother textures. Among the unaged samples, monolithic EPDM showed the best performance: lowest abrasion loss, highest tensile strength (0.93 MPa), and greatest elongation (52.4%), owing to its compact structure and smooth finish.

In contrast, the spray-coated surface exhibited the weakest durability. Despite maintaining tensile strength (~0.83 MPa), it showed extensive cracking, increased porosity, and roughness after aging, leading to performance failures under wet conditions. The sandwich system offered intermediate behavior—initially the weakest (0.57 MPa), it gained strength post-UV exposure, though at the cost of flexibility and surface integrity. Its rough surface maintained acceptable grip in wet conditions.

Cycle 1 caused moderate degradation, while Cycle 2 intensified erosion and mechanical fatigue, especially in spray-coated systems. The results emphasize that binder content, surface texture, and filler cohesion are key factors in long-term performance.

Key conclusions include the following:

- Monolithic EPDM is most durable and suitable for high-performance use due to its superior mechanical and aging resistance.
- Spray-coated systems are vulnerable to environmental damage and less suitable for long-term outdoor use.
- Sandwich-type systems offer a good compromise between grip and durability, particularly in wet conditions.
- Water spray aging more closely replicates real-world wear, reinforcing the need for robust material design.
- Surface roughness and tensile changes are critical indicators of degradation and should complement traditional tests.
- Future improvements should target binder optimization, filler dispersion, and use of stabilizers/nanomaterials, supported by realistic standardized aging protocols.

## Figures and Tables

**Table 1:** Slip resistance measurements under dry conditions.

	Slip Resistance [PTV]		
	Spray-Coated Dual-Layer	Monolithic EPDM	Sandwich-Type Dual-Layer Surface
**Location 1**	74; 75; 75; 75; 74	82; 81; 82; 80; 80	77; 76; 79; 76; 78
**Location 2**	76; 74; 75; 76; 76	83; 82; 84; 84; 82	77; 79; 76; 75; 75
**Location 3**	77; 77; 75; 76; 76	84; 85; 83; 83; 84	77; 79; 77; 80; 78
**Mean value**	**75**	**83**	**77**

**Table 2:** Slip resistance measurements under wet conditions.

	Slip Resistance [PTV]		
	Spray-Coated Dual-Layer	Monolithic EPDM	Sandwich-Type Dual-Layer Surface
**Location 1**	50; 51; 51; 49; 50	55; 56; 55; 56; 57	61; 60; 62; 60; 61
**Location 2**	52; 51; 52; 53; 52	57; 55; 56; 55; 56	62; 62; 63; 61; 62
**Location 3**	53; 51; 52; 53; 52	55; 54; 55; 55; 56	63; 63; 61; 62; 62
**Mean value**	**51**	**56**	**62**

**Table 3:** Slip resistance measurements after aging (Cycle 1: UV + condensation)—dry conditions.

	Slip Resistance [PTV]		
	Spray-Coated Dual-Layer	Monolithic EPDM	Sandwich-Type Dual-Layer Surface
**Location 1**	68; 68; 70; 71; 69	70; 72; 71; 70; 72	74; 73; 75; 75; 74
**Location 2**	67; 69; 68; 67; 69	71; 69; 72; 71; 70	72; 74; 73; 72; 72
**Location 3**	70; 69; 71; 71; 70	69; 68; 70; 70; 71	73; 71; 72; 71; 72
**Mean value**	**69**	**71**	**73**

**Table 4:** Slip resistance measurements after aging (Cycle 1: UV + condensation)—wet conditions.

	Slip Resistance [PTV]		
	Spray-Coated Dual-Layer	Monolithic EPDM	Sandwich-Type Dual-Layer Surface
**Location 1**	65; 64; 64; 65; 64	62; 61; 62; 63; 63	65; 66; 65; 65; 66
**Location 2**	64; 65; 63; 53; 64	61; 62; 62; 61; 62	67; 67; 66; 68; 67
**Location 3**	67; 66; 64; 65; 65	63; 62; 62; 61; 61	65; 65; 64; 66; 65
**Mean value**	**64**	**62**	**66**

**Table 5:** Slip resistance measurements after aging (Cycle 2: UV + water spray)—dry conditions.

	Slip Resistance [PTV]		
	Spray-Coated Dual-Layer	Monolithic EPDM	Sandwich-Type Dual-Layer Surface
**Location 1**	72; 73; 74; 72; 72	71; 72; 72; 71; 73	76; 75; 75; 75; 76
**Location 2**	74; 73; 71; 72; 71	72; 70; 71; 73; 71	74; 75; 73; 75; 75
**Location 3**	71; 70; 70; 71; 71	70; 71; 70; 71; 72	76; 76; 74; 74; 74
**Mean value**	**72**	**71**	**75**

**Table 6:** Slip resistance measurements after aging (Cycle 2: UV + water spray)—wet conditions.

	Slip Resistance [PTV]		
	Spray-Coated Dual-Layer	Monolithic EPDM	Sandwich-Type Dual-Layer Surface
**Location 1**	61; 60; 62; 61; 62	57; 57; 57; 58; 59	66; 65; 65; 65; 64
**Location 2**	63; 62; 61; 61; 62	60; 59; 58; 59; 59	66; 64; 64; 65; 64
**Location 3**	60; 60; 62; 61; 62	58; 60; 57; 58; 57	66; 67; 67; 66; 66
**Mean value**	**61**	**58**	**65**

**Table 7:** Summary of slip resistance measurements before and after aging using both methods.

Tested Parameter	Type of Surface								
	Spray-Coated Dual-Layer			Monolithic EPDM			Sandwich-Type Dual-Layer		
	Reference	Cycle 1	Cycle 2	Reference	Cycle 1	Cycle 2	Reference	Cycle 1	Cycle 2
**Slip resistance in dry conditions**	75	69	72	83	71	71	77	73	75
**Slip resistance in wet condition**	51	64	61	56	62	58	62	66	65
**Standard deviation**	3.01	2.92	2.01	2.81	3.05	2.08	2.43	2.86	2.86
	2.51	1.73	1.12	1.15	1.92	1.76	1.21	1.85	1.19
***p*-Value**	0.22	0.18	0.23	0.23	0.26	0.29	0.31	0.26	0.23
	0.43	0.6	0.16	0.15	0.06	0.23	0.23	0.12	0.14

Expanded uncertainty of slip resistance measurement (related to the accuracy of the measuring equipment): U = 3 PTV (at an approximately 95% confidence level with a coverage factor k = 2).

**Table 8:** Summary of abrasion resistance test results for synthetic sports surfaces.

Sample	Mass Loss [g]		
	Spray-Coated Dual-Layer	Monolithic EPDM	Sandwich-Type Dual-Layer
**Reference**	0.9026	1.4261	2.5150
**Cycle 1**	0.8377	1.1910	2.4654
**Cycle 2**	0.7454	1.0557	2.3572
**Standard deviation**	0.08	0.18	0.42
	0.05	0.21	0.53
	0.08	0.17	0.47
***p*-Value**	0.012	0.21	0.35
	0.052	0.26	0.31
	0.041	0.24	0.32

**Table 9:** Summary of tensile properties measurements before and after aging using both methods.

Tested Parameter	Type of Surface								
	Spray-Coated Dual-Layer			Monolithic EPDM			Sandwich-Type Dual-Layer		
	Reference	Cycle 1	Cycle 2	Reference	Cycle 1	Cycle 2	Reference	Cycle 1	Cycle 2
**Tensile strength [MPa]**	0.83	0.84	0.83	0.93	0.98	0.89	0.57	0.82	0.75
**Elongation at maximum force [%]**	50.9	38.7	41.6	52.4	53.3	46.2	37.8	45.3	45.7

Expanded uncertainty of slip resistance measurement (related to the accuracy of the measuring equipment): U = 0.01 MPa; U = 0.5% (at an approximately 95% confidence level with a coverage factor k = 2).

**Table 10:** Surface roughness results.

Sample Name	Microstructure of Sample	S_a_ [µm]
Spray-coated dual-layer_reference	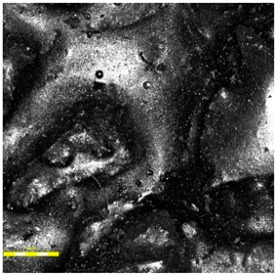	431.08
Spray-coated dual-layer _cycle_1	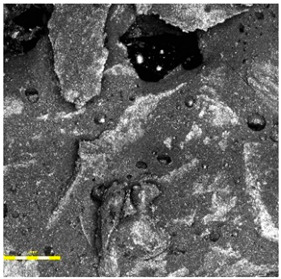	543.65
Spray-coated dual-layer _cycle_2	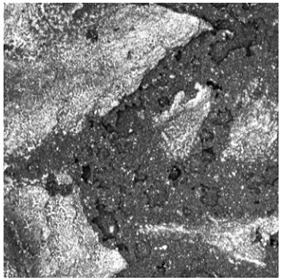	367.84
Sandwich-type dual-layer_reference	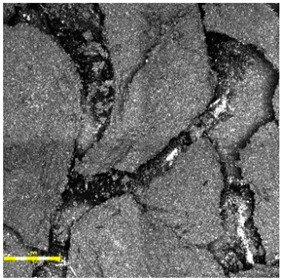	746.86
Sandwich-type dual-layer _cycle_1	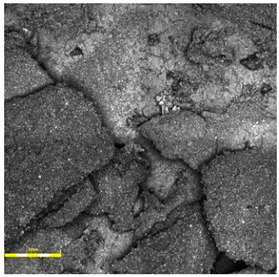	831.32
Sandwich-type dual-layer _cycle_2	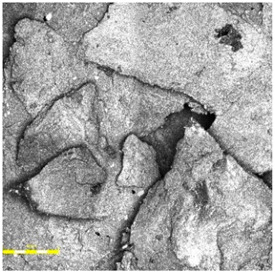	521.53
Monolithic EPDM_reference	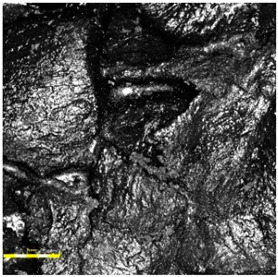	287.90
Monolithic EPDM _cycle_1	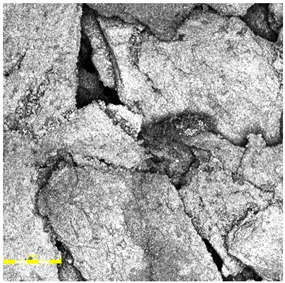	426.54
Monolithic EPDM _cycle_2	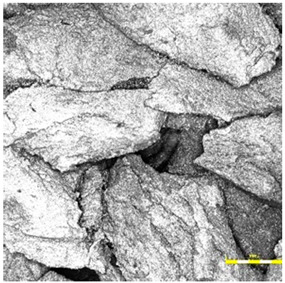	329.45

## Data Availability

The original contributions presented in this study are included in the article and Supplementary Materials. Further inquiries can be directed to the corresponding author.

## Supplementary Materials

The following supporting information can be downloaded at: https://www.mdpi.com/article/10.3390/ma18143342/s1, Table S1. Surface roughness results—3D reconstruction maps.

## References

[1] Yuan Z., Li W., Li C., Ye L. (2019). Construction of multiple crosslinking networks in EPDM rubber: Synergistic reinforcing effect of graphene-zinc dimethacrylate on EPDM and improvement mechanism of sealing resilience. Compos. Part A Appl. Sci. Manuf..

[2] Liu Q., Li J., Jiang Y., Cong C., Xu L., Zhang Y., Meng X., Zhou Q. (2021). Effect of crosslinked structure on the chemical degradation of EPDM rubber in an acidic environment. Polym. Degrad. Stab..

[3] Azizi S., Momen G., Ouellet-Plamondon C., David E. (2020). Performance improvement of EPDM and EPDM/silicone rubber composites using modified fumed silica, titanium dioxide and graphene additives. Polym. Test..

[4] Salimi A., Abbassi-Sourki F., Karrabi M., Ghoreishy M.H.R. (2021). Investigation on viscoelastic behavior of virgin EPDM/reclaimed rubber blends using Generalized Maxwell Model (GMM). Polym. Test..

[5] Wachtendorf V., Kalbe U., Krüger O., Bandow N. (2017). Influence of weathering on the leaching behaviour of zinc and PAH from synthetic sports surfaces. Polym. Test..

[6] Tagliabue S., Andena L., Pavan A., Marenghi A., Testa E., Frassine R. (2018). Ageing in athletics tracks: A multi-technique experimental investigation. Polym. Test..

[7] Andena L., Tagliabue S., Pavan A., Marenghi A., Testa M., Frassine R. (2020). Probing athletics tracks degradation using a microscratch technique. Polym. Test..

[8] Hong S., Park N., Ju S., Lee A., Shin Y., Kim J.S., Um M.-K., Yi J.W., Chae H.G., Park T. (2023). Molecular degradation mechanism of segmented polyurethane and life prediction through accelerated aging test. Polym. Test..

[9] Al Maamori M.H., Kateeb M. (2016). Effect of weathering conditions on the mechanical properties of the sport surfaces prepared of crumb rubber. Int. J. ChemTech Res..

[10] Kim S., Shin H.-O., Yoo D.-Y. (2020). Mechanical and dynamic behavior of an elastic rubber layer with recycled styrene-butadiene rubber granules. Polymers.

[11] Strąk C., Sudoł E. (2021). Odporność na zużycie zewnętrznych syntetycznych nawierzchni sportowych. Mater. Bud..

[12] Strąk C., Małek M., Jackowski M., Sudoł E. (2021). Safety comes first: Novel Styrene Butadiene Rubber (SBR) and Ethylene Propylene Diene Monomer (EPDM) surfaces as a response to sport injuries. Materials.

[13] Małek M., Jackowski M., Strąk C., Sudoł E. (2020). Analysis of the impact of artificial aging on the mechanical properties of synthetic sports surfaces. J. Civil Eng. Manag..

[14] Sudoł E., Strąk C. (2019). Durability of synthetic sports surfaces under various environmental conditions. Mater. Bud..

[15] Kaczmarek J., Nowak P. (2018). Analysis of the technological parameters’ influence on the quality of asphalt sports field pavements. Bud. i Inżynieria Sr..

[16] Zieliński M., Kowalski R. (2019). Modern materials in the construction of sports pavements. Mater. Bud..

[17] Kowalczyk A., Wiśniewski S. (2020). Durability assessment of polyurethane pavements on sports facilities. Przegląd Bud..

[18] Szymański P., Lewandowski T. (2017). Comparative studies of synthetic and natural pavements on football fields. Inżynieria i Bud..

[19] Wójcik J., Adamczak M. (2016). Evaluation of the operating costs of sports pavements made of different materials. Ekon. i Sr..

[20] Pawlak Z., Nowicki P. (2015). Manufacturing technologies of sports pavements made of ecological materials. Build. Technol..

[21] Kaczmarek L., Zieliński P. (2014). The influence of weather conditions on the technical condition of sports field pavements. Bud. i Archit..

