# Supplementary material for: Degradation of the Surface of Synthetic Layered Composites Due to Accelerated Ageing
Source: Materials (Basel). 2025 Jul 16;18(14):3342. doi: 10.3390/ma18143342 (PMC12299265; doi:10.3390/ma18143342)
Supplement: Supplementary file 1 [file materials-18-03342-s001.zip › materials-3702671-supplementary.pdf]

materials

Table S1. Surface roughness results – 3D reconstruction maps.

Sample name	3D map
Spray-coated dual-layer_ref-erence	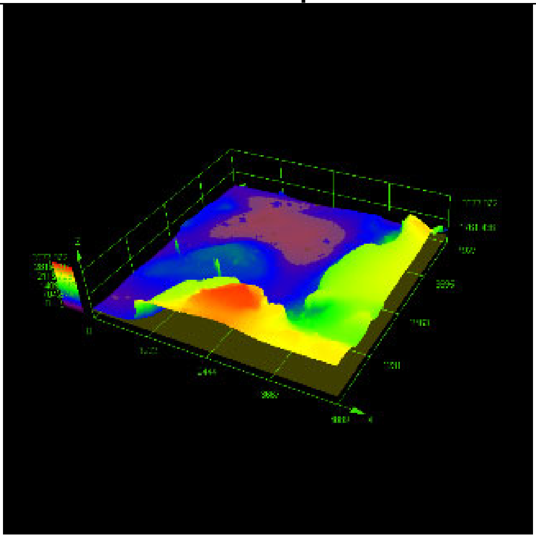
Spray-coated dual-layer _cy-cle_1	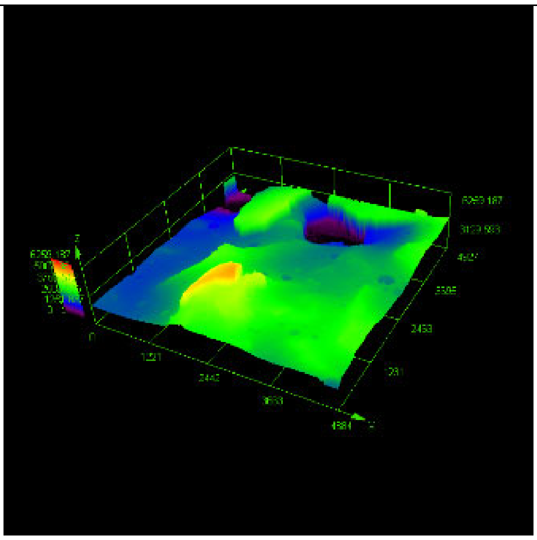

Spray-coated dual-layer _cy-cle_2	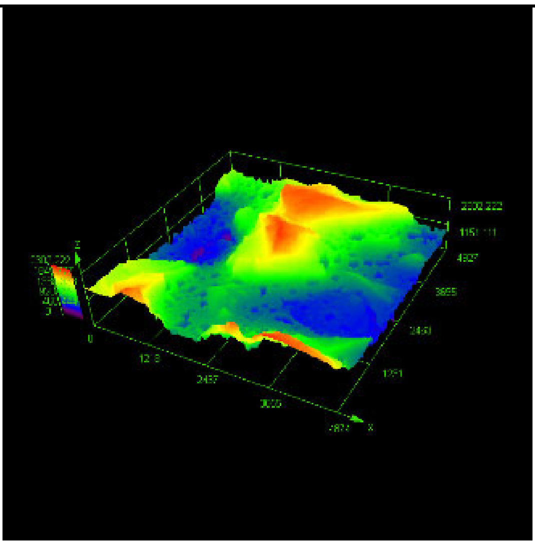
Sandwich-type dual-layer_reference	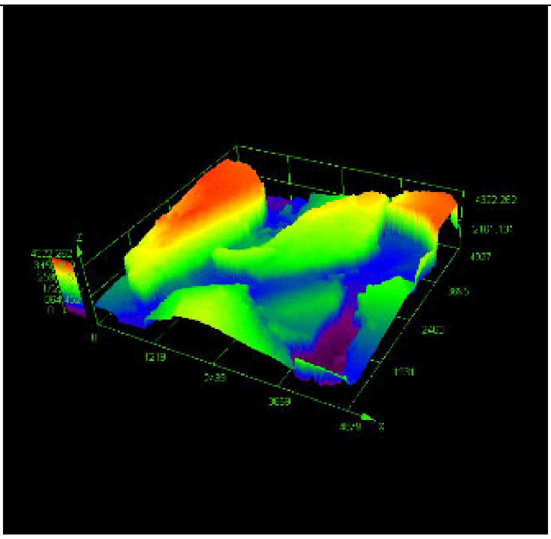
Sandwich-type dual-layer _cycle_1	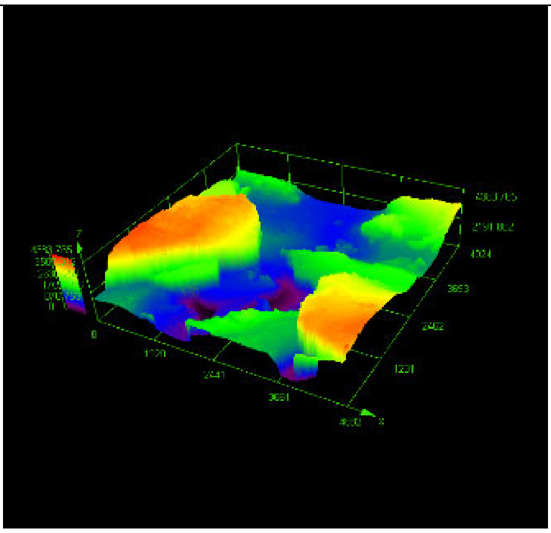

Sandwich-type dual-layer _cycle_2	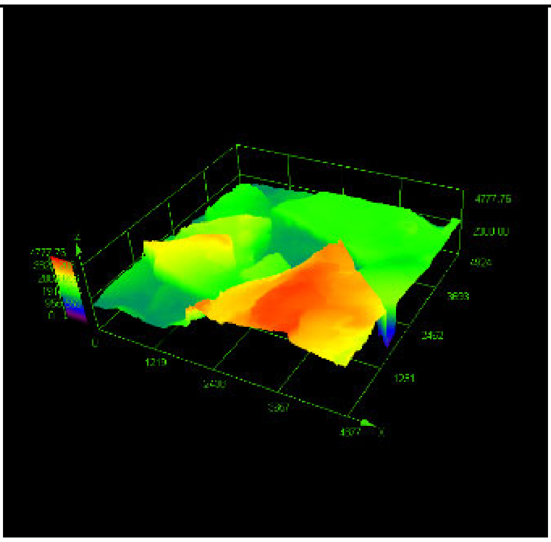
Monolithic EPDM_reference	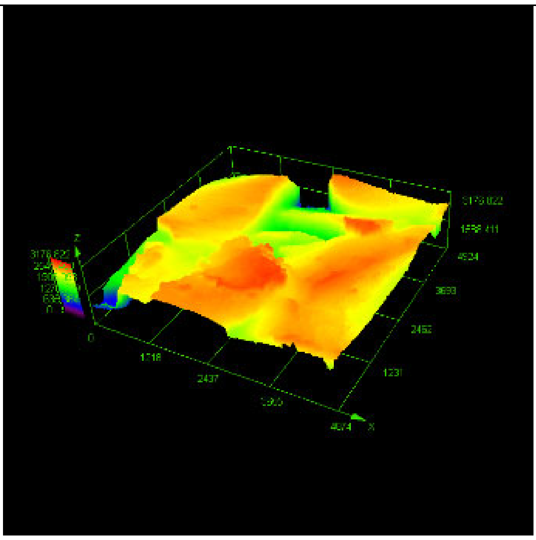
Monolithic EPDM _cycle_1	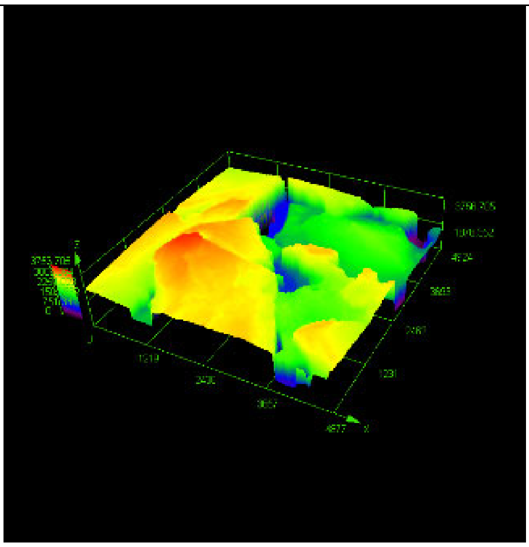

Monolithic EPDM _cycle_2	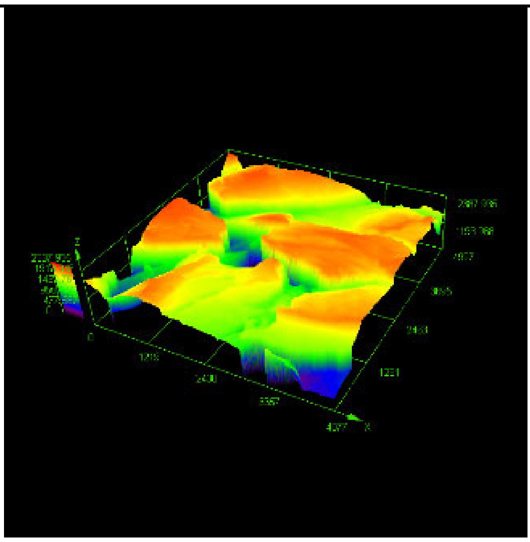
